# Identification of a Five-mRNA Signature as a Novel Potential Prognostic Biomarker for Glioblastoma by Integrative Analysis

**DOI:** 10.3389/fgene.2022.931938

**Published:** 2022-07-08

**Authors:** Huifang Xu, Linfang Zhang, Xiujuan Xia, Wei Shao

**Affiliations:** ^1^ Department of Neurology, Wuhan No. 1 Hospital, Huazhong University of Science and Technology, Wuhan, China; ^2^ Department of Gastroenterology, The Third Xiangya Hospital, Central South University, Changsha, China

**Keywords:** glioblastoma, prognosis, signature, survival, biomarker

## Abstract

Despite the availability of advanced multimodal therapy, the prognosis of patients suffering from glioblastoma (GBM) remains poor. We conducted a genome-wide integrative analysis of mRNA expression profiles in 302 GBM tissues and 209 normal brain tissues from the Gene Expression Omnibus (GEO), The Cancer Genome Atlas (TCGA), and the Genotype-Tissue Expression (GTEx) project to examine the prognostic and predictive value of specific mRNAs in GBM. A total of 26 mRNAs were identified to be closely related to GBM patients’ OS (*p* < 0.05). Utilizing survival analysis and the Cox regression model, we discovered a set of five mRNAs (PTPRN, ABCC3, MDK, NMB, and RALYL) from these 26 mRNAs that displayed the capacity to stratify patients into high- and low-risk groups with statistically different overall survival in the training set. The model of the five-mRNA biomarker signature was successfully verified on a testing set and independent sets. Moreover, multivariate Cox regression analysis revealed that the five-mRNA biomarker signature was a prognostic factor for the survival of patients with GBM independent of clinical characteristics and molecular features (*p* < 0.05). Gene set enrichment analysis indicated that the five-mRNA biomarker signature might be implicated in the incidence and development of GBM through its roles in known cancer-related pathways, signaling molecules, and the immune system. Moreover, consistent with the bioinformatics analysis, NMB, ABCC3, and MDK mRNA expression was considerably higher in four human GBM cells, and the expression of PTPRN and RALYL was decreased in GBM cells (*p* < 0.05). Our study developed a novel candidate model that provides new prospective prognostic biomarkers for GBM.

## Background

Glioblastoma (GBM), also referred to as glioblastoma multiforme, is a grade IV glioma that is the most aggressive type of brain cancer with a high morbidity and mortality rate, accounting for 15% of all brain tumors (Batash et al., 2017; Ostrom et al., 2019). The prognosis and treatment of GBM are very poor because many kinds of cell types are involved. Every year, a large number of people are affected, and the survival duration ranges from 8 to 15 months (Anjum et al., 2017). In patients who had surgery, chemotherapy, and radiation treatment, the median survival period with GBM was 15–16 months (Alifieris and Trafalis, 2015). Unfortunately, only a few minor advances in the prognosis of GBM patients were made in the last decade. Thereby, understanding the molecular mechanisms and developing effective biomarkers to predict prognosis is critical for GBM patients (Polivka et al., 2017; Sasmita et al., 2018).

GBM development is a complex process involving numerous gene alterations. A thorough examination of the molecular mechanisms underlying GBM is critical for the diagnosis and treatment of GBM patients. High-throughput sequencing technologies have been commonly applied with the rapid development of genomics, and several data can be freely accessed from public databases including the Gene Expression Omnibus (GEO), The Cancer Genome Atlas (TCGA), the Genotype-Tissue Expression (GTEx) project, and ArrayExpress. Numerous research studies on brain gene expression profiles have been conducted in recent years utilizing these open database platforms, and these studies have revealed hundreds of differentially expressed genes (DEGs) of GBM that may be implicated in the formation and progression of GBM (Fatai and Gamieldien, 2018; Han and Puri, 2018; Qian et al., 2018). Several investigations utilizing high-throughput sequencing revealed the results of gene expression signatures in GBM. However, the common drawback of gene expression profiling studies is batch effects with the combat function due to many factors, including the application of different microarray and sequencing platforms, different data processing methods, small sample sizes, and different backgrounds of samples (Stein et al., 2015; Yi et al., 2018). To overcome limitations resulting from batch effects in a single-cohort study, we applied surrogate variable analysis (SVA), which is an unbiased approach to integrate multiple data sources and remove batch effects. It has been demonstrated that removing batch effects and using surrogate variables reduces dependence, stabilizes error rate estimates, and improves reproducibility (Leek et al., 2012).

The development of accurate tools to predict the prognosis of GBM patients is of crucial importance to clinical diagnosis and treatment decisions. In this investigation, the integrated bioinformatics strategy was employed to systematically examine the prognostic value of mRNAs in GBM patients from the GEO, TCGA, and GTEx databases. Cox regression analysis and the risk score model technique were used to develop a biologically relevant five-mRNA signature capable of predicting the prognosis of GBM patients in the training set. The prognostic value of the five-mRNA signature was first confirmed in large GBM samples from different databases. Furthermore, these five mRNA expressions were closely associated with immune microenvironment regulation, ERBB signaling pathway, and MAPK signaling pathway in the development of GBM. These findings not only provide reliable independent prognostic factors but also expand our knowledge of the function of these five mRNAs in the development and progression of GBM.

## Methods

### Microarray Datasets

**FIGURE 1:**
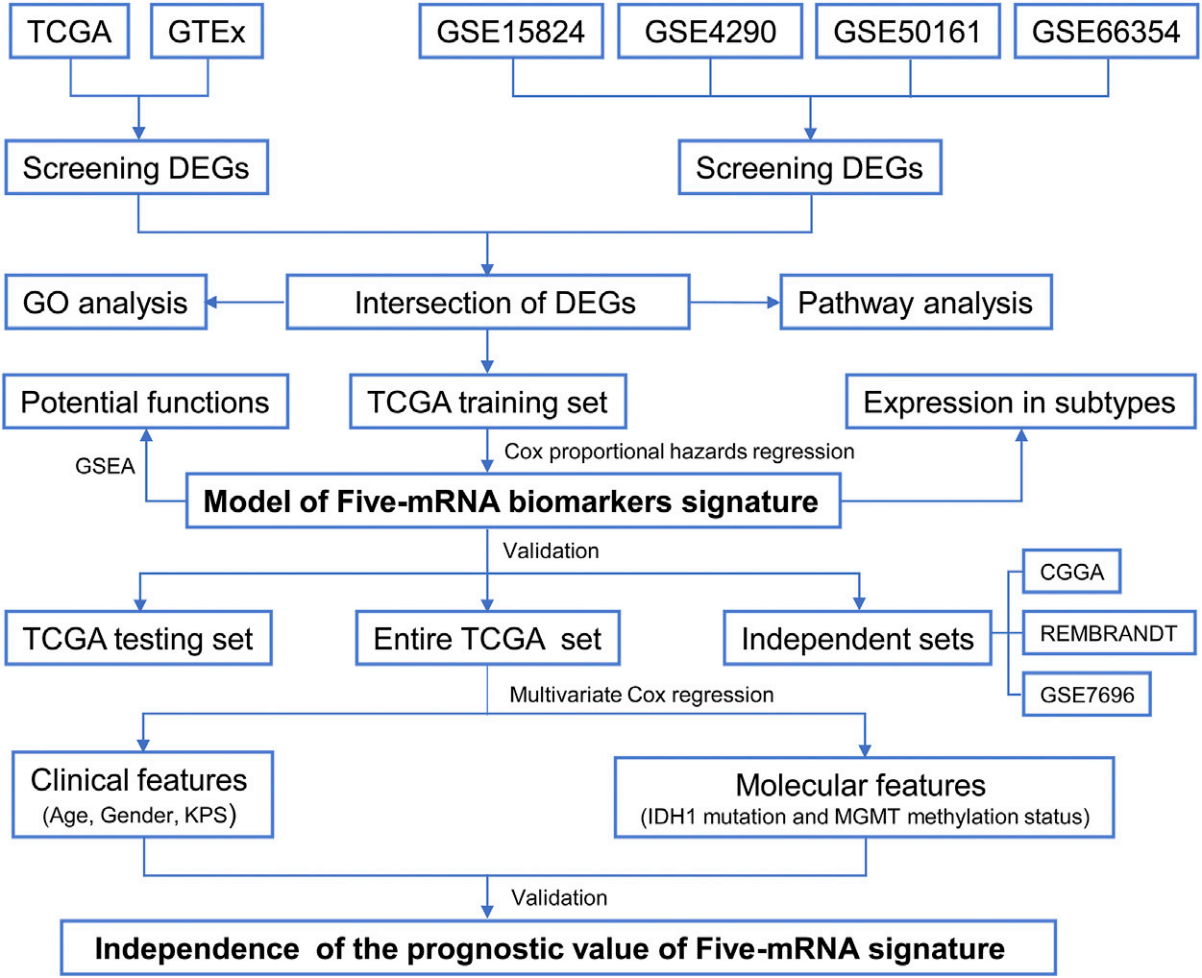
Flow chart of the study.

The GEO database was used to obtain gene expression profiles of GSE4290, GSE50161, GSE15824, and GSE66354 from GBM and normal brain tissue. These four series, which included 142 GBM tissues and 51 normal brain tissues, were built using the GPL570 platform (Affymetrix Human Genome U133 Plus 2 Array, Affymetrix, Santa Clara, CA, United States). These four datasets were selected for integrated analysis since they share the same platform, which is essential for merging data from multiple datasets. The downloaded files of raw data from the four gene chips were processed using the R software package. Calibration, standardization, and log2 transformation were performed on the data. The gene expression profiles of these four datasets were combined for the analysis, and the robust multiarray average was utilized to preprocess the CEL files (Bolstad et al., 2003). To eliminate the batch effects of these four datasets, the combat function in the SVA package was used (Leek et al., 2012). Table 1 displays the data information, and Figure 1 depicts the flow chart of our investigation.

**TABLE 1:** Information for GBM data.

Sample	Database	Platform	GBM	Normal
Brain	GSE4290	GPL570	77	23
Brain	GSE50161	GPL570	34	13
Brain	GSE15824	GPL570	12	2
Brain	GSE66354	GPL570	19	13
Brain	TCGA_GBM	Illumina HiSeq 2000	160	5
Brain	GTEx	Illumina HiSeq 2000	0	153
Total			302	209

### GTEx RNA Sequencing Dataset

The GTEx (release V7) project provided the RNA expression profiles of 1,426 normal brain tissue samples (https://www.gtexportal.org/home/). The expression data of 153 brain samples were randomly chosen and quantified as raw read counts.

### TCGA RNA Sequencing and Clinical Datasets

The RNA expression profiles (RNA-Seq2 level 3 data, platform: Illumina HiSeq2000 RNA sequencing, through August 2019) of 160 GBM tissues and five normal brain tissues were extracted from the TCGA data repository (https://portal.gdc.cancer.gov). Meanwhile, clinical data from those 160 GBM patients were extracted. Verhaak et al.’s (2010) study provided information on the molecular features and subtypes of GBM patients. Using the ‘sample’ function from the R package, the 160 GBM patients from the TCGA database were randomly assigned to a training set (*n* = 80) and a testing set (*n* = 80). Table 2 lists the detailed clinical features of all GBM sets. The gene expression profiles from GTEx and TCGA were integrated using the robust multiarray average and normalized by DESeq2. The SVA package was used to remove the batch effect.

**TABLE 2:** Clinical and molecular features of GBM patients.

Characteristic		Training set (*n* = 80)	Testing set (*n* = 80)	TCGA set (*n* = 160)
Age				
	≤60	39	43	82
	>60	41	37	78
Gender				
	Male	46	58	104
	Female	34	22	56
Vital status				
	Alive	13	17	30
	Dead	67	63	130
KPS				
	≤70	40	34	74
	>70	40	46	86
				
Subtype				
	Classical	21	18	39
	Mesenchymal	24	28	52
	Neural	20	8	28
	Proneural	14	24	38
	Unknown	1	2	3
IDH status				
	Mutant	4	5	9
	Wild type	72	71	143
	Unknown	4	4	8
MGMT status				
	Methylated	30	26	56
	Unmethylated	31	36	67
	Unkown	19	18	37

### Identification of DEGs

The DEGs in GBM and normal brain tissue samples from the four integrated microarray datasets, GTEx data, and TCGA data, were assessed by the limma package (*p* ≤ 0.05, log_2_ fold change (logFC) ≥ 2, false discovery rate (FDR) ≤ 0.01). Afterward, the intersections of DEGs from the four integrated microarray datasets and GTEx-TCGA data were identified and used for further bioinformatics analyses.

### Functional Enrichment Analysis

Metascape was used to conduct Gene Ontology (GO) and Kyoto Encyclopedia of Genes and Genomes (KEGG) analysis on the DEGs (http://www.metascape.org/). GO was utilized to describe gene function from three aspects: molecular functions (MFs), cellular components (CCs), and biological processes (BPs). KEGG analysis was employed to examine the signaling pathways involved in the DEGs. In addition, gene set enrichment analysis (GSEA) was performed to evaluate the correlation between DEG expression and cancer-related pathways. The GSEA protocol is detailed on the Broad Institute Gene Set Enrichment Analysis website (http://www.broad.mit.edu/gsea).

### Survival Analysis

Univariate Cox proportional hazards regression analysis was conducted to determine the DEGs and clinical features that were closely related to overall survival (OS). The genes and clinical characteristics with log-rank *p*-values less than 0.05 were then employed in a multivariate Cox proportional hazards regression analysis to identify prognosis-related genes. In addition, the least absolute shrinkage and selection operator (LASSO) estimation-based Cox-PH model was applied to determine the specific prognosis-related genes by the penalized package in the R language.

A risk score model for predicting the prognosis of GBM patients was developed by incorporating the expression level of each optimal prognostic mRNA weighted by their regression coefficient from the multivariate Cox regression model (Li et al., 2021) shown as follows:

$$\text{Risk score }\left(\text{patient}\right)=\sum_{i}\text{coefficient}\left(\text{mRNA}i\right)\times \text{expression}\left(\text{mRNA}i\right),$$

where mRNA i  is the candidate of the i th selected mRNA. The risk score model is a measure of the prognostic risk for each GBM patient. All samples in the training set were separated into two groups: high-risk (risk score greater than the median) and low-risk (risk score less than the median). Moreover, the reliability and validity of the risk score model were verified in independent sets, including the REMBRANDT study, Chinese Glioma Genome Atlas (CGGA) database, and GSE7696. The log-rank test and Kaplan–Meier survival analysis were used to compare the OS times of the high-risk and low-risk groups. Hazard ratios (HRs) and 95% confidence intervals (CIs) were assessed. The sensitivity and specificity of the prognostic prediction model were compared using receiver operating characteristic (ROC) curve analysis. The area under the curve (AUC) was also determined.

### Cell Culture and Quantitative Real-Time PCR

Human normal glial cell line HEB and human glioblastoma cell lines (A172, LN299, U118, and U138) were cultured in DMEM medium with 10% FBS at 37°C in a humidified incubator with 5% CO2. The cells were harvested during their logarithmic growth phase and their total RNA was extracted using the Trizol reagent (Invitrogen, Carlsbad, CA, United States). To extract cDNA, reverse transcription was conducted using a reverse transcription kit (TaKaRa, Dalian, China) following the manufacturer’s instructions. The relative levels of mRNA were measured by qRT-PCR. The sequences of the specific primers utilized in this study are shown in Supplementary Table S1. Comparative quantification was performed using the 2^−ΔΔCt^ method, with target gene expression normalized to GAPDH.

### Statistical Analysis

R studio (version 3.5.1) and SPSS 20.0 were used for statistical analysis (SPSS Inc., Chicago, IL, United States). Differentially expressed mRNAs were determined using the limma package in R studio. Student’s t-test (two-tailed) and the Kruskal–Wallis test were used to compare the difference between two groups or more than two groups, respectively. When the *p*-value was less than 0.05, differences were deemed statistically significant.

## Results

### Identification of DEGs in the Four Microarray Datasets and TCGA Dataset

**FIGURE 2:**
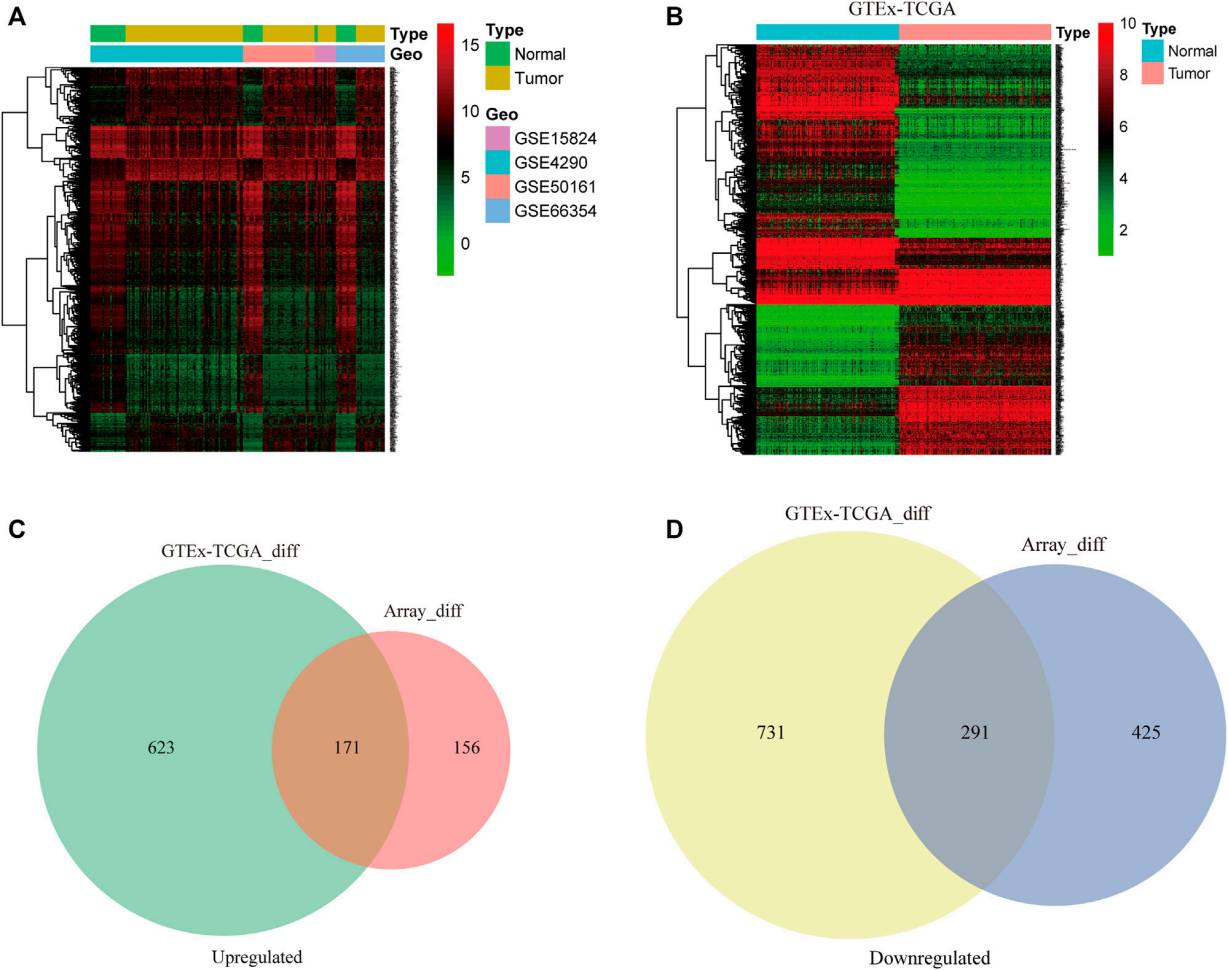
The differentially expressed genes (DEGs) from multiple datasets are analyzed. The DEGs in glioblastoma (GBM) and normal brain tissue samples from the four integrated microarray datasets **(A)** and Genotype-Tissue Expression (GTEx)-The Cancer Genome Atlas (TCGA) datasets **(B)** were analyzed by the limma package and are shown in the hierarchical clustering heatmap. Venn diagram analysis of the intersections of the upregulated genes **(C)** and downregulated genes **(D)** from four microarray datasets and the GTEx-TCGA datasets.

**FIGURE 3:**
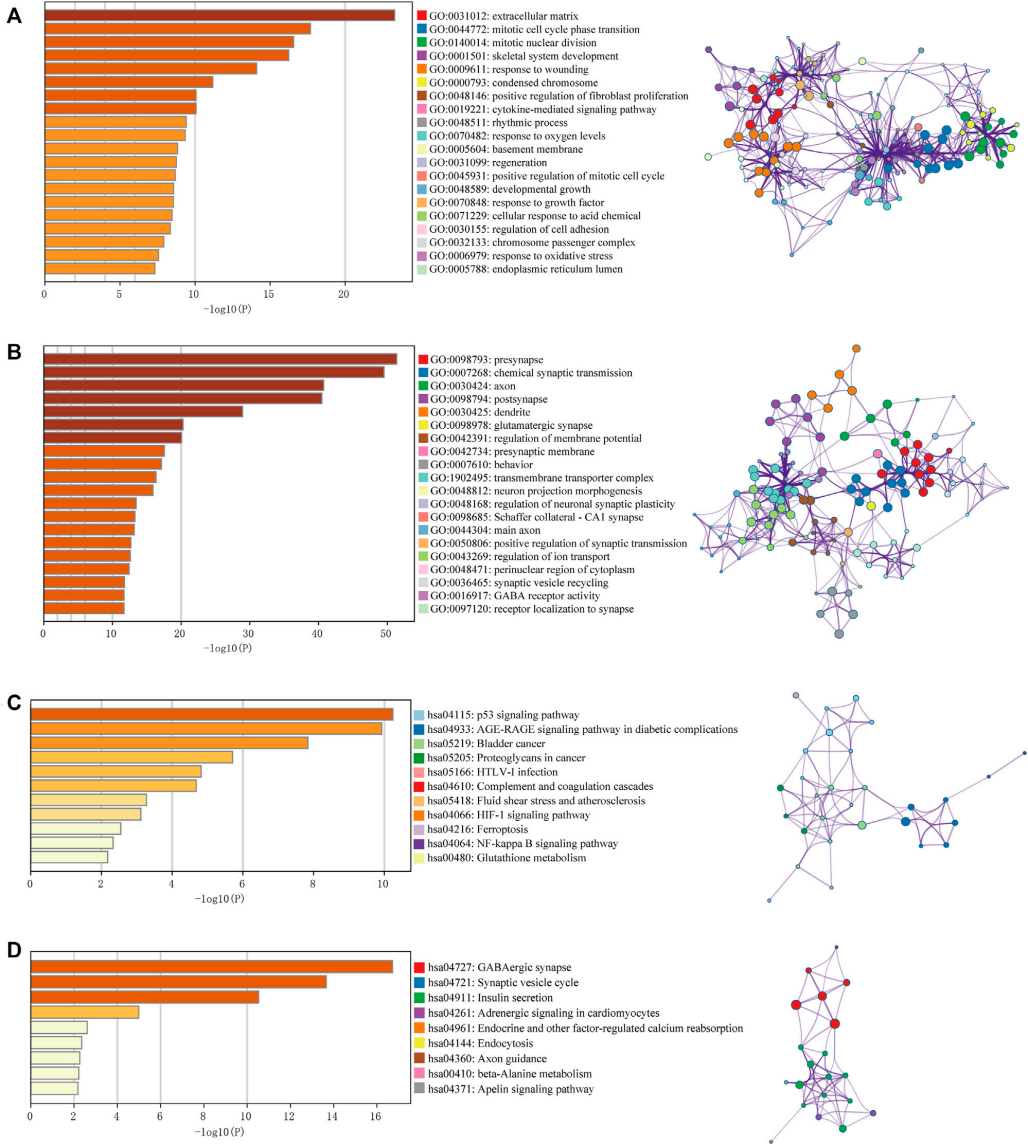
Gene Ontology (GO) term and Kyoto Encyclopedia of Genes and Genomes (KEGG) pathway analysis for DEGs. GO results of the intersecting upregulated genes **(A)** and downregulated genes **(B)**. KEGG results of the intersecting upregulated genes **(C)** and downregulated genes **(D)**. The relationships among the enriched clusters from the GO and KEGG analyses were visualized with Metascape (http://www.metascape.org/).

The raw data from the four microarray datasets were integrated for analysis. The data information is shown in Table 1. The robust multiarray average algorithm and combat function of the SVA package was employed to preprocess and eliminate the batch effects of these integrated data. When the integrated data was evaluated using the limma package (*p* ≤ 0.05, logFC ≥ 2, FDR ≤ 0.01), 1,043 DEGs were detected, comprising 327 upregulated genes and 716 downregulated genes. To further study whether these genes are differentially expressed between normal brain tissue and GBM tissue, we analyzed the DEGs in 318 brain tissue samples from the TCGA and GTEx databases, including 160 GBM samples and 158 normal brain tissue samples. We identified 794 significantly upregulated mRNAs and 1,022 downregulated mRNAs. The DEGs are shown on the heat map in Figures 2A,B based on the |logFC| value. Afterward, we detected 462 intersecting mRNAs from the integrated microarray data and GTEx-TCGA data (171 upregulated and 291 downregulated), as shown in Figures 2C,D. We used Metascape to conduct GO and KEGG pathway enrichment analysis to investigate the potential roles of these dysregulated mRNAs. The GO terms in which the upregulated genes were enriched were mostly extracellular matrix, mitotic cell cycle phase transition, and developmental growth (Figure 3A), while the primary roles of the downregulated mRNAs involved pre-synapse, chemical synaptic transmission, and regulation of neuronal synaptic plasticity (Figure 3B). The p53 signaling pathway, HIF-1 signaling route, and NF-kappa B signaling pathway were the most significant KEGG pathways in which the elevated genes were enriched (Figure 3C). The GABAergic synapse, synaptic vesicle cycle, and apelin signaling pathway were all related to the downregulated mRNAs (Figure 3D). These results indicate that most of the dysregulated mRNAs participate in carcinogenesis and the development of GBM through modulating BPs and critical pathways.

### Identification of Prognosis-Related Genes From the TCGA Training Set

The 160 GBM patients from the TCGA database were assigned randomly to the training sample set (*n* = 80) and the testing sample set (*n* = 80), as shown in Table 2. To determine the prognosis-related genes, the expression data of the DEGs were analyzed by univariate Cox proportional hazards regression analysis in the training set. A total of 26 mRNAs were identified to be closely related to GBM patients’ OS (*p* < 0.05) and were thus involved in the candidate pool for further multivariate Cox proportional hazards regression analysis to analyze their independent prognostic value. According to the Cox model, five of the 26 candidate genes were discovered to be independent biomarkers for prognosis in GBM. Among the five prognostic mRNAs, three mRNAs (NMB, ABCC3, and MDK) with positive coefficients may be prognostic risk factors, and their high expression was correlated with shorter survival, while the remaining two mRNAs (PTPRN and RALYL) with negative coefficients tended to be protective factors, and their high expression was correlated with longer survival (Table 3).

**TABLE 3:** Five mRNAs selected as prognosis-associated factors in GBM.

Gene symbol	Ensembl ID	Coefficient	HR	95% CI of HR		*p* value	Gene expression associated with poor prognosis
				Lower	Upper		
PTPRN	ENSG00000054356	0.3340	1.397	1.099	1.775	0.006	High
NMB	ENSG00000197696	-0.1793	0.836	0.671	1.041	0.110	Low
RALYL	ENSG00000184672	-0.3547	0.701	0.498	0.988	0.042	Low
ABCC3	ENSG00000108846	0.1849	1.203	0.975	1.485	0.085	High
MDK	ENSG00000110492	0.4231	1.527	1.101	2.117	0.011	High

### The Five-mRNA Prognostic Risk Model and Predictability Assessment

**FIGURE 4:**
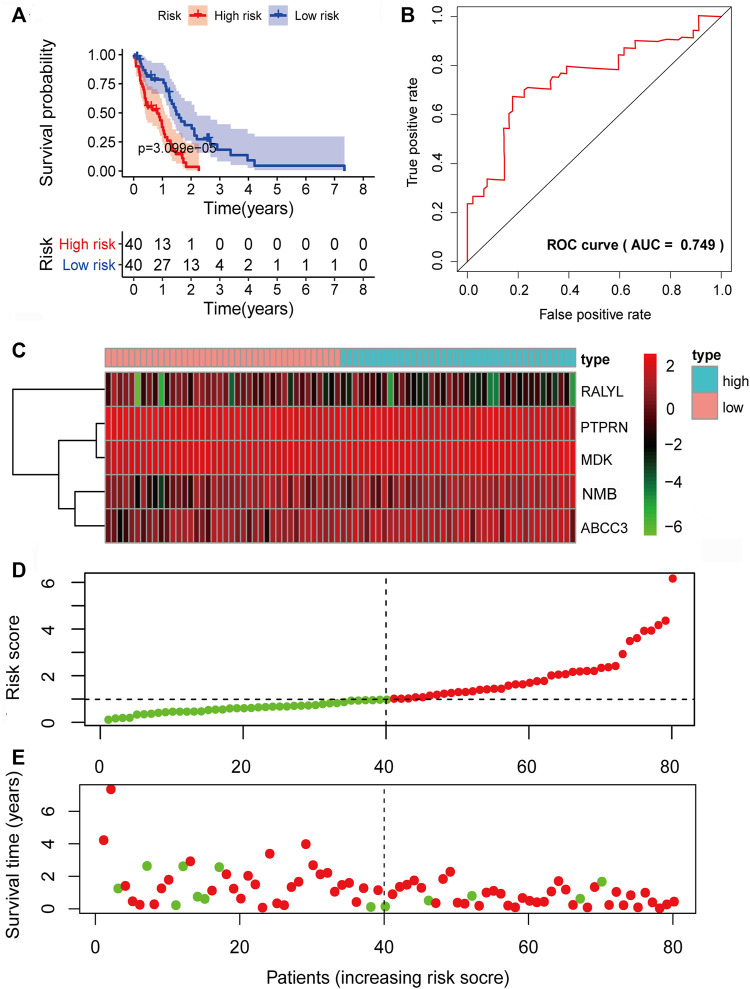
Prognostic evaluation of the five-mRNA signature in the training set. **(A)** Kaplan–Meier survival curve for patient overall survival (OS) in the training set. **(B)** Receiver operating characteristic (ROC) curve analysis to compare the sensitivity and specificity of the prognosis prediction model. The mRNA expression patterns **(C)**, risk score distribution **(D),** and survival status of patients **(E)** in the high- and low-risk groups by the five-mRNA signature. Green dot, alive; red dot, dead.

Given the significant and independent association between the expression of the five prognosis-associated mRNAs and OS, the five prognosis-associated mRNAs were combined to construct a five-mRNA biomarker signature to predict the prognosis of the patients. The risk score model was constructed according to the regression coefficients from the multivariate Cox regression model as follows: risk score= (−0.1793×PTPRN expression) + (0.3340×NMB expression) + (−0.3547×RALYL expression) + (0.1849×ABCC3 expression) + (0.4231×MDK expression). Based on the risk score model, the five-mRNA prognostic risk score for each GBM patient in the training set was calculated. According to the median risk score, all patients in the training set were divided into two groups: high risk (*n* = 40) and low risk (*n* = 40). Kaplan–Meier survival analysis was performed to compare the OS of the two risk groups of patients in the training set (Figure 4A). The median survival time for the high-risk group was shorter than that of the low-risk group (0.8219 years vs. 1.4575 years, *p* = 3.099e-05, log-rank test). The high-risk group had lower 2-year survival rates than those in the low-risk group (3.630% vs. 36.48%, *p* < 0.001). The prognostic power of the five-mRNA biomarker signature was assessed by computing the AUC of the ROC curve. The ROC curve analysis revealed an AUC of 0.749, indicating that the five-mRNA biomarker signature model has good sensitivity and specificity in predicting GBM patient survival risk (Figure 4B). The heat map showed the expression patterns of the five prognosis-associated mRNAs between the high-risk group and low-risk group. For patients with low-risk scores, the expression levels of the two protective mRNAs were upregulated and those of the three risk mRNAs were downregulated. The expression of the five prognosis-associated mRNAs, on the other hand, showed the reverse patterns in patients with high-risk scores (Figure 4C). The risk score distribution and the survival status of the GBM patients in the training set are marked on the dot plot shown in Figures 4D, E, respectively.

**FIGURE 5:**
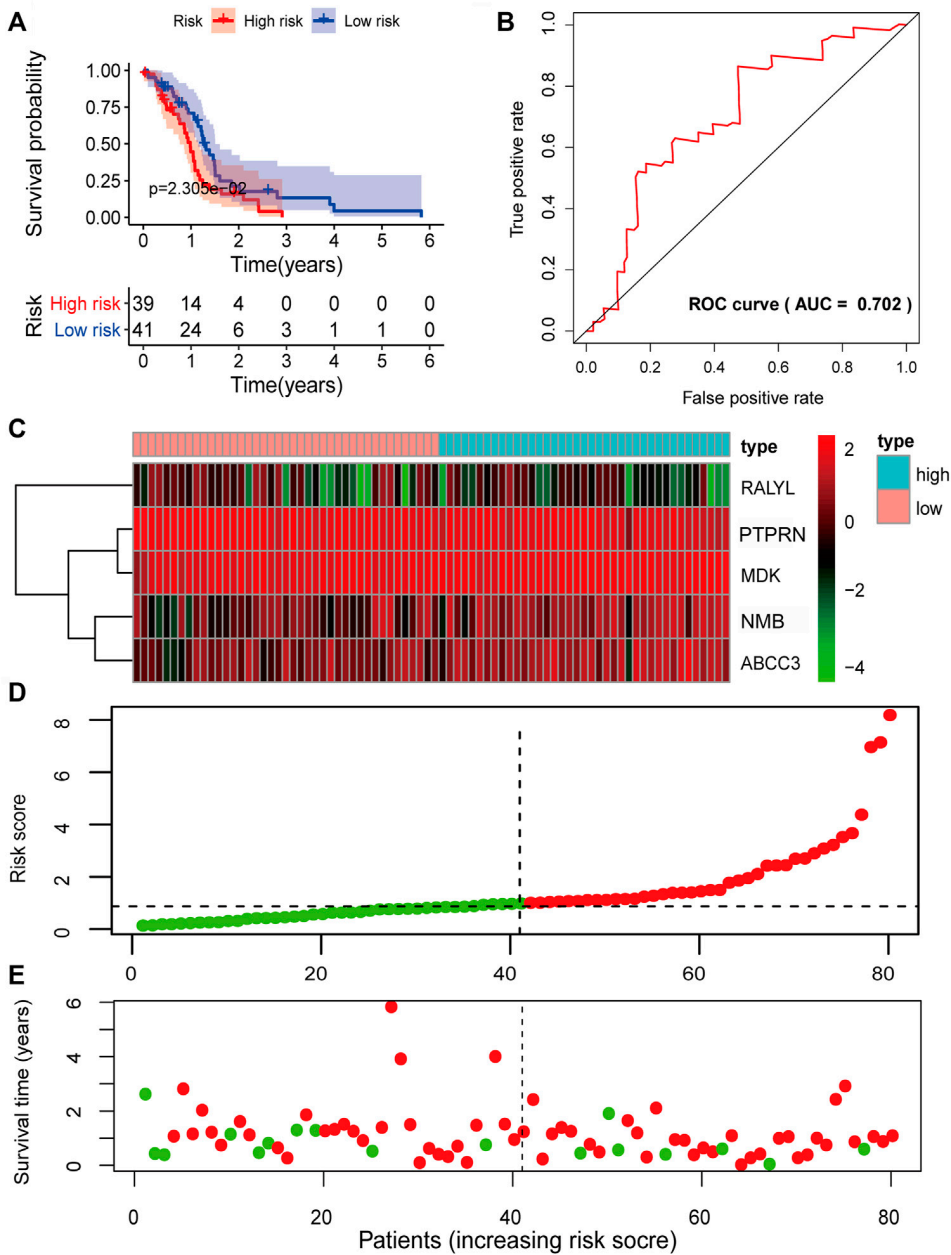
Prognostic evaluation of the five-mRNA signature in the testing set. **(A)** Kaplan–Meier survival curve for patient overall survival (OS) in the testing set. **(B)** Receiver operating characteristic (ROC) curve analysis to compare the sensitivity and specificity of the prognosis prediction model. The mRNA expression patterns **(C)**, risk score distribution **(D),** and survival status of patients **(E)** in the high- and low-risk groups by the five-mRNA signature. Green dot, alive; red dot, dead.

**FIGURE 6:**
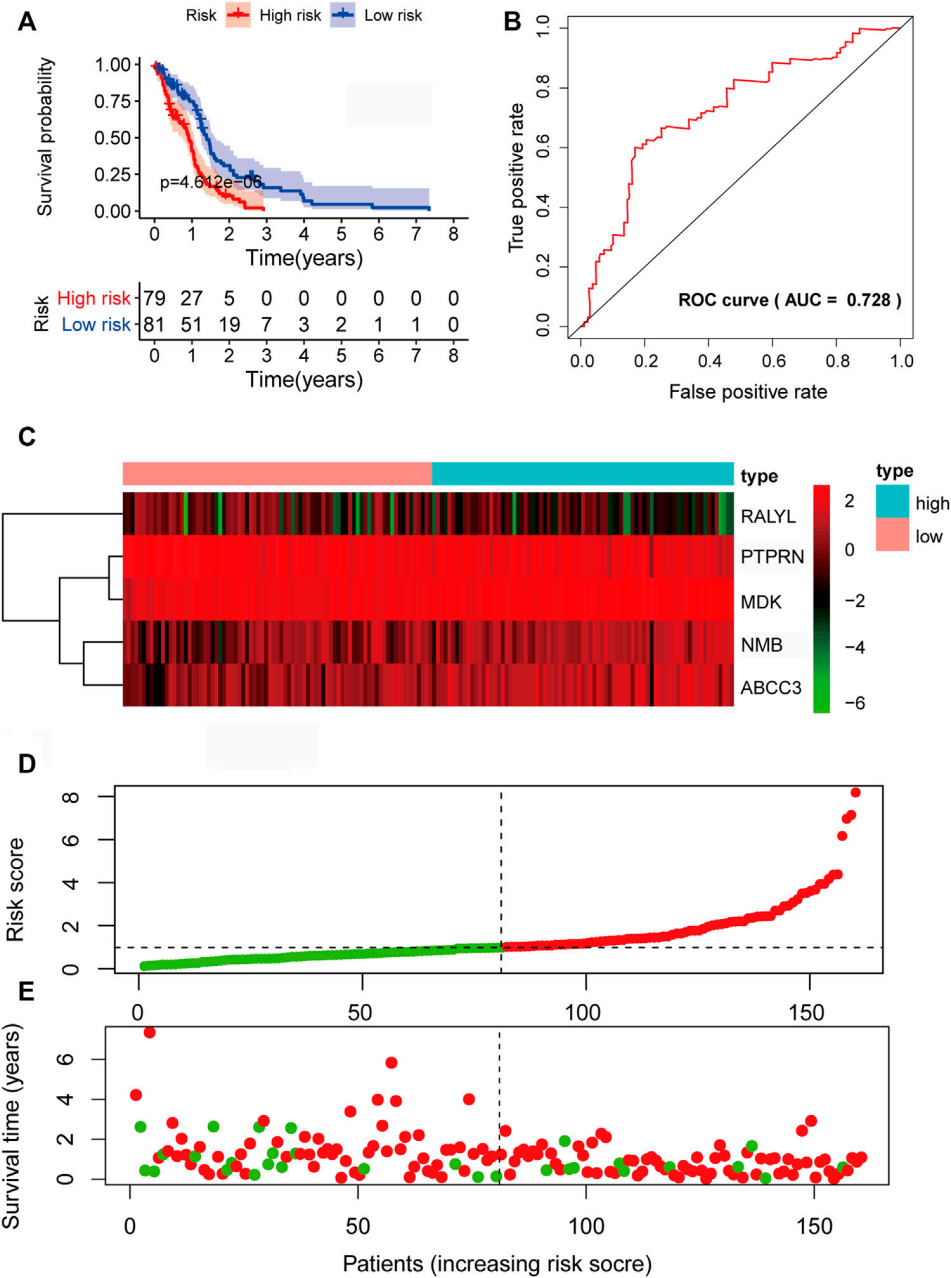
Prognostic evaluation of the five-mRNA signature in The Cancer Genome Atlas (TCGA) set. **(A)** Kaplan–Meier survival curve for patient overall survival (OS) in the entire TCGA set. **(B)** Receiver operating characteristic (ROC) curve analysis to compare the sensitivity and specificity of the prognosis prediction model. The mRNA expression patterns **(C)**, risk score distribution **(D),** and survival status of patients **(E)** in the high- and low-risk groups by the five-mRNA signature. Green dot, alive; red dot, dead.

To verify the predictive power of the biomarker signature, we computed the five-mRNA signature-based risk scores of 80 patients in the testing set. The patients from the testing set were also divided into high-risk groups and low-risk based on the same median cutoff point obtained from the training set (median survival: 0.9370 years vs. 1.2466 years, *p* = 2.305e-02, log-rank test). The high-risk group had 2-year survival rates of approximately 11.95% vs. 17.68% in the low-risk group (*p* < 0.001) (Figure 5A). The AUC value was 0.702 (Figure 5B). The expression patterns of the five prognosis-associated mRNAs (Figure 5C) were similar to the results of the training set. Figures 5D,E demonstrate the distribution of risk scores and the survival status of GBM patients. In the overall TCGA set, the performance of predicting patient prognosis by the five-mRNA signature was consistent with the aforementioned results. Kaplan–Meier analysis showed that the median survival times of the high-risk group and the low-risk group were 0.9123 and 1.3863 years (*p* = 4.612e-05), respectively. The 2-year survival rates for the high-risk and low-risk groups were 8.14% and 27.65% (*p* < 0.001), respectively (Figure 6A). The AUC value was 0.728 (Figure 6B). Figures 6C–E depict the expression patterns of the five prognosis-associated mRNAs, the risk score distribution, and the survival status of GBM patients.

**FIGURE 7:**
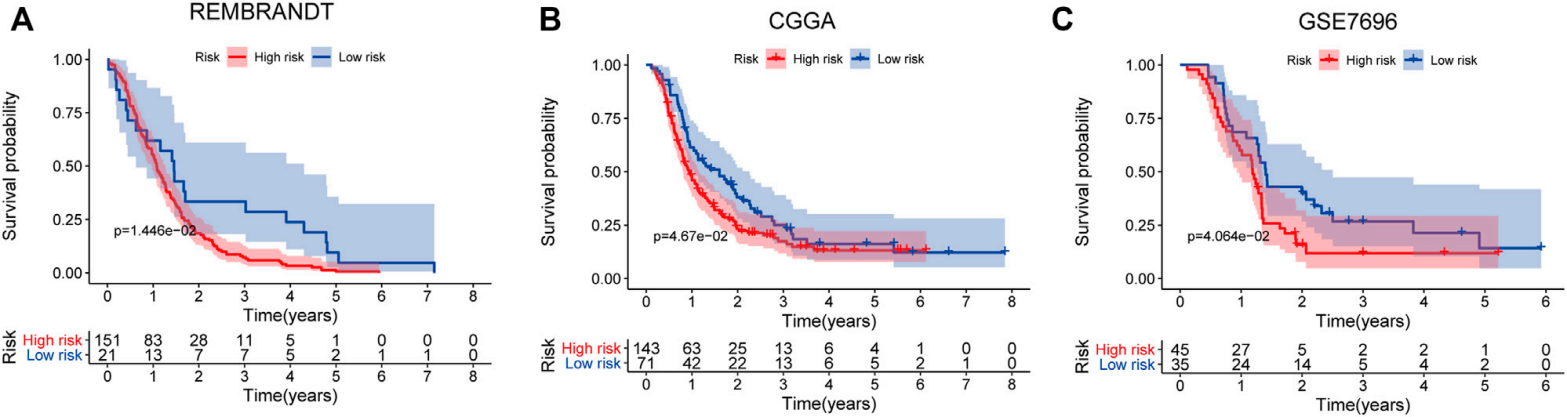
Survival prediction of the five-mRNA signature in the independent sets. Kaplan–Meier survival curve of overall survival (OS) between high- and low-risk patients in the REMBRANDT study **(A)**, Chinese Glioma Genome Atlas (CGGA) datasets **(B),** and GSE7696 dataset **(C)**.

The prognostic value of the five-mRNA signature was confirmed using independent sets, including the REMBRANDT study, CGGA database, and GSE7696, to further evaluate its robustness. By comparing the patient’s risk score to the cutoff determined from the training set, each patient in the independent sets was also categorized as a high-risk or low-risk case. The log-rank test demonstrated that there was a statistically different OS between the low-risk group and the high-risk group in these independent sets (*p* < 0.05). Consistent with the findings of the training set described earlier, the five-mRNA biomarker signature model was found to be a predictive factor for the prognosis of GBM (Figures 7A–C).

### Independence of the Prognostic Value of the Five-mRNA Signature From Clinical Variables and Molecular Features

**FIGURE 8:**
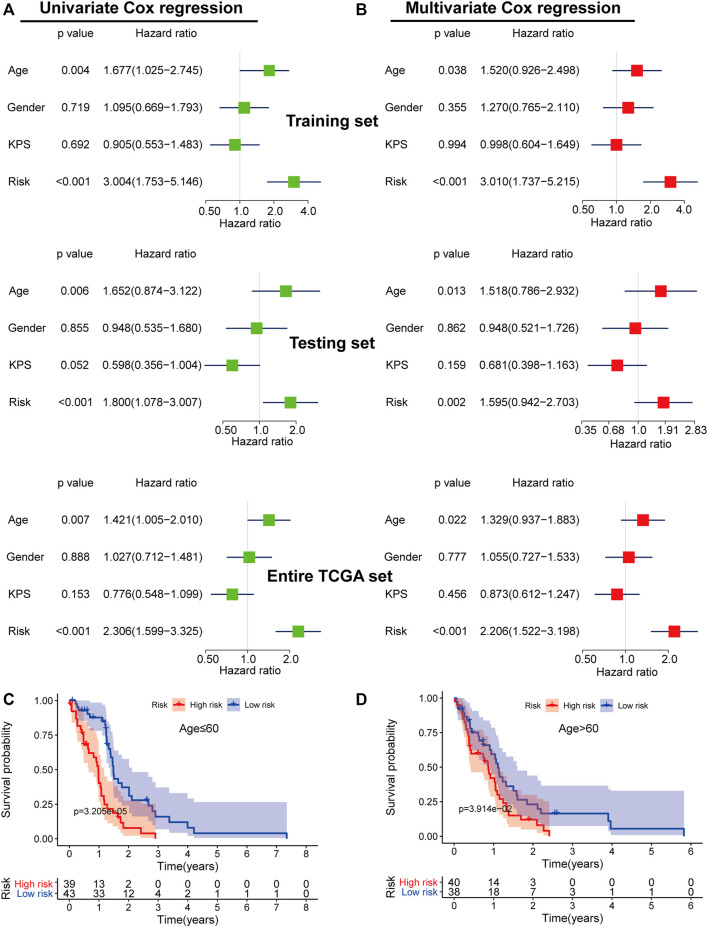
The independence of the prognostic value of the five-mRNA signature from clinical characteristics. Univariate **(A)** and multivariate **(B)** Cox regression analyses of the correlation between GBM patient overall survival (OS) and clinical characteristics (age, gender, and Karnofsky Performance Score (KPS)). Kaplan–Meier survival curve analysis of OS in the high- and low-risk groups for young patients (≤ 60 years old) **(C)** and old patients (> 60 years old) **(D)**.

To determine if the five-mRNA signature was a prognostic factor independent of other clinical features, we conducted univariable and multivariable Cox regression analyses using the five-mRNA signature risk score and clinical features as covariates (age, gender, Karnofsky performance score (KPS)) (Figures 8A, B). Multivariable Cox regression analysis results demonstrated that the five-mRNA signature was closely correlated with OS in each set (training set, testing set, and entire TCGA set) when adjusting for other clinical features (Figure 8B). We also found that age was an independent predictor of OS in GBM patients. As a result, stratification analysis was carried out to investigate the age dependence of the five-mRNA signature. Using the five-mRNA signature, patients of each age group (young patient group: age ≤ 60, *n* = 82; old patient group: age > 60, *n* = 78) were categorized into two groups: low-risk and high-risk. The log-rank test demonstrated that there was a statistically different OS between the low-risk group and the high-risk group (*p* = 3.205e-05 for the young patient group and *p* = 3.941e-02 for the old patient group) in each age group (Figures 8C, D).

**FIGURE 9:**
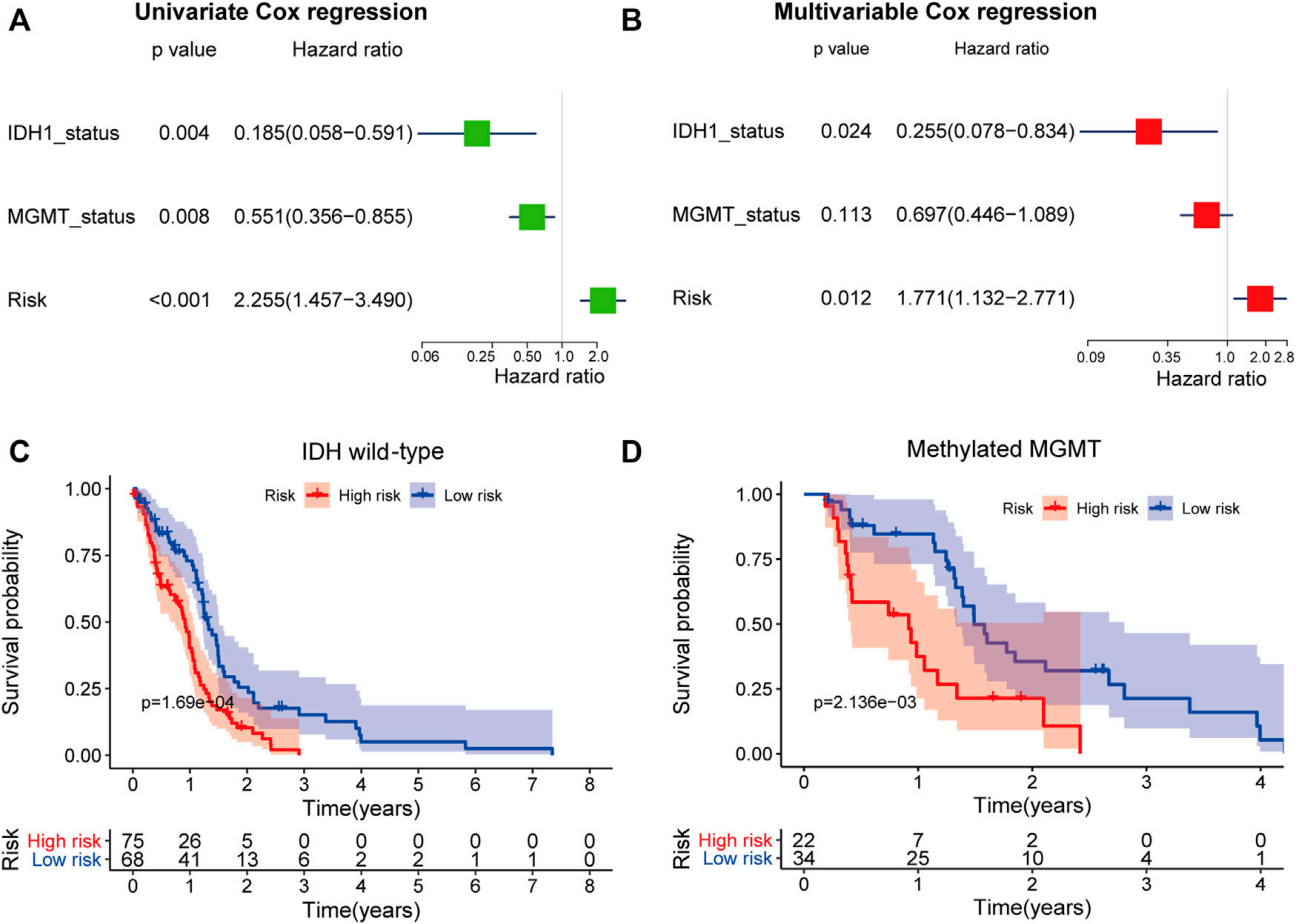
The independence of the prognostic value of the five-mRNA signature from molecular features. Univariate **(A)** and multivariate **(B)** Cox regression analyses of the correlation between GBM patient overall survival (OS) and clinical characteristics (IDH1 mutation and MGMT promoter methylation status). Kaplan–Meier survival curve analysis of OS in the high- and low-risk groups for patients with IDH wild type **(C)** and methylated MGMT status **(D)**.

Furthermore, we used univariable Cox regression (Figure 9A) and multivariable Cox regression (Figure 9B) analyses to investigate if the predictive power of the five-mRNA signature for survival was independent of other observed prognostic factors, including IDH1 mutation and MGMT promoter methylation status. The results showed that the five-mRNA signature was substantially linked with survival when adjusted for IDH1 mutation and MGMT promoter methylation status, indicating that the five-mRNA signature’s predictive potential for GBM survival is also independent of these two molecular features (Figure 9B). Interestingly, we also discovered that IDH1 status was closely related to the OS. Therefore, we classified the GBM patients in this study into two groups (IDH1 wild-type group, *n* = 143; IDH1 mutation group, *n* = 8) and investigated whether the five-mRNA signature was able to predict the survival of patients. The results indicated that there was a significantly different OS between the low-risk group and the high-risk group (*p* = 1.69e-04) in the IDH1 wild-type patients (Figure 9C), suggesting that the five-mRNA signature could determine a subgroup of IDH1 wild-type patients who had a better prognosis. Although the multivariable Cox regression analysis results indicated that MGMT status (methylated MGMT group, *n* = 56; unmethylated MGMT, *n* = 67) was not significantly correlated with OS (*p* > 0.05), the five-mRNA signature was also able to determine a subgroup of methylated MGMT patients who had a higher chance of survival (Figure 9D).

### The Expression Levels of the Five-mRNA Signature in the Subtypes of GBM

**FIGURE 10:**
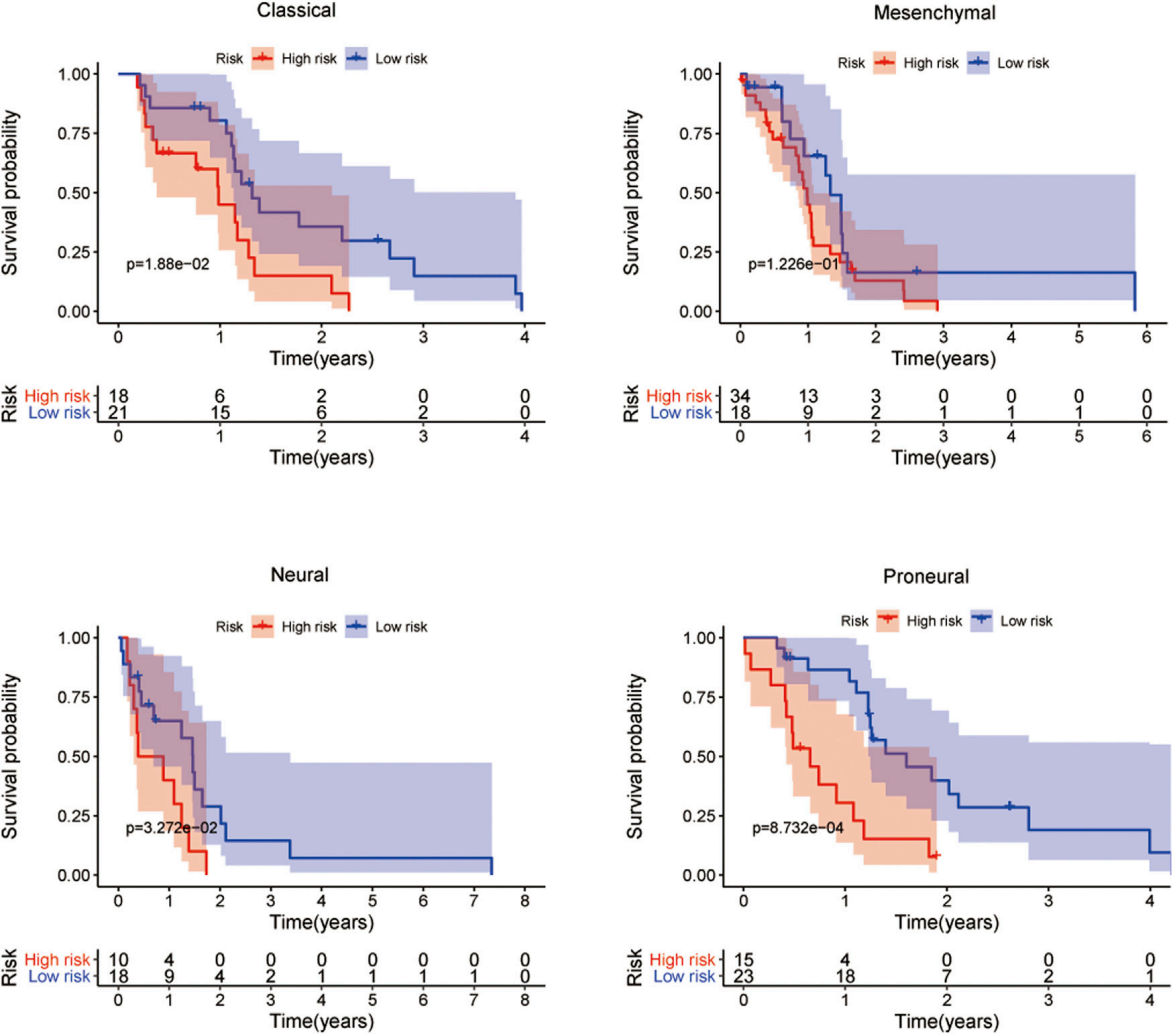
The prognostic value of the five-mRNA signature in the subtypes of GBM. Kaplan–Meier survival curve analysis of OS in the high- and low-risk groups for patients with the four subtypes. Kruskal–Wallis test was used to compare the expression levels of each mRNA in the four subtypes of GBM.

Next, we investigated the expression levels of the five-mRNA signature in four GBM subtypes (classical, mesenchymal, neural, and proneural). The findings demonstrated a significant difference in the distribution of all five mRNA expression levels across the four GBM subtypes, demonstrating that the five-mRNA signature is also a subtype-specific marker (Supplementary Figure S1). The Kaplan–Meier survival analysis of OS revealed a substantial difference between the high-risk and low-risk groups of patients with three different subtypes (classical, neural, and proneural). These findings suggest that the five-mRNA signature is an independent prognostic factor for OS in GBM patients of various subtypes (Figure 10).

### Functional Characterization of the Five-mRNA Signature in GBM

**FIGURE 11:**
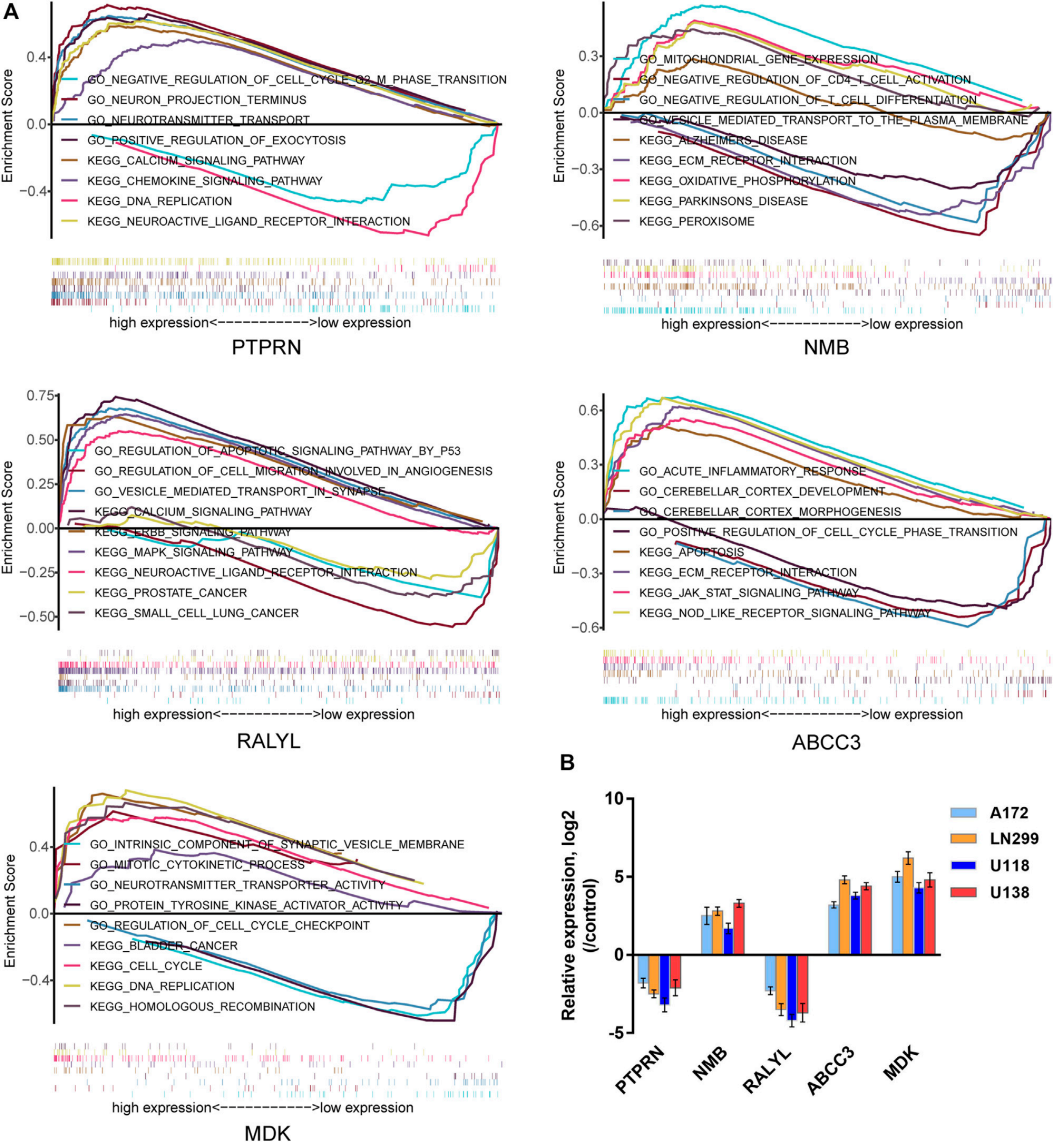
The potential biological function of the five-mRNA signature in GBM. **(A)** Gene set enrichment analysis (GSEA) using stratified five-mRNA signature expression levels for genes downregulated or upregulated in GBM. The GSEA results showed the correlation between the five-mRNA levels and potential biological functions in the Gene Ontology (GO) and Kyoto Encyclopedia of Genes and Genomes (KEGG) databases. **(B)** The relative expression levels of the five-mRNA signature were determined by qRT-PCR in four human GBM cell lines.

Using the TCGA GBM data, we conducted GSEA to provide new insights into the functions of the five-mRNA signature. The subjects were sorted from low to high according to the expression level of the five mRNAs, and the TCGA GBM data were loaded into R studio and analyzed with the GSEA package. Stratified expression levels of the five-mRNA signature were closely related to genes associated with the occurrence and development of GBM, such as the regulation of cell cycle and cell apoptosis, brain development, immune response, MAPK signaling pathway, and ERBB signaling pathway (Figure 11A). Next, we performed a co-expression network analysis following the Pearson correlation coefficient (|cor| ≥ 0.55, *p* < 0.01) in the entire TCGA dataset to further reveal the potential biological functions of the five-mRNA signature in GBM. A total of 762 protein-coding genes (PCGs) were found to be strongly associated with at least one mRNA in the five-mRNA signature. The potential function of all PCGs associated with the five-mRNA signature was then predicted using enrichment analysis based on GO terms and KEGG pathways. Consistent with the GSEA results, the results from GO and KEGG analyses revealed that the five-mRNA signature may be involved in cell morphogenesis involved in neuron differentiation, brain development, the apoptosis pathway, the MAPK signaling pathway, the Ras signaling pathway, and the ERBB signaling pathway (Table 4).

**TABLE 4:** GO and KEGG analyses of the protein targets of the five-mRNA signature.

Items	Category ID	Log_10_P
GO		
Cell morphogenesis involved in neuron differentiation	GO:0048667	−12.85
Brain development	GO:0007420	−8.10
Acute inflammatory response	GO:0002526	−5.12
Humoral immune response	GO:0006959	−2.97
Astrocyte cell migration	GO:0043615	−3.00
Apoptotic signaling pathway	GO:0097190	−2.80
KEGG		
MAPK signaling pathway	hsa04010	−6.23
Neuroactive ligand–receptor interaction	hsa04080	−10.20
Ras signaling pathway	hsa04014	−4.95
TNF signaling pathway	hsa04668	−4.45
ERRB signaling pathway	hsa04012	−3.43
ECM-receptor interaction	hsa04512	−2.37

GO, Gene Ontology; KEGG, Kyoto Encyclopedia of Genes and Genomes.

### Experimental Verification of Expression Levels of the Five-mRNA Signature in GBM Cell Lines

Finally, we validated the expression of the five-mRNA signature in four human GBM cell lines (A172, LN299, U118, and U138) and human normal glial cell line HEB using qRT-PCR. As revealed in Figure 11B, NMB, ABCC3, and MDK mRNA expression were considerably higher in GBM cells than in the control group (HEB cells). Conversely, the expression of PTPRN and RALYL was decreased in GBM cells (*p* < 0.05). The findings were consistent with the bioinformatics analysis outlined earlier.

## Discussion

In this article, we gathered four series from the GEO database and integrated datasets from the TCGA and GTEx databases to conduct an integrative analysis in order to thoroughly examine the data and identify relevant gene markers. In the DEG analysis, we found 171 upregulated and 291 downregulated DEGs after combining the data from the four GEO datasets and GTEx-TCGA datasets. The GO and KEGG pathway analyses of the 362 aberrantly expressed mRNAs revealed the crucial BPs and pathways in GBM, most of which were classic pathways and BPs that play important roles in GBM, such as extracellular matrix, regulation of the mitotic cell cycle, the p53 signaling pathway, the HIF-1 signaling pathway, and the NF-kappa B signaling pathway. Interestingly, some novel BPs and pathways involved in GBM progression and development, including chemical synaptic transmission, regulation of neuronal synaptic plasticity, GABA receptor activity, and the apelin signaling pathway. Following that, we investigated the relationship between these 362 aberrantly expressed mRNAs and prognosis in GBM patients by performing a genome-wide analysis of the 362 aberrantly expressed mRNAs in 80 patients in the training set and discovered 26 mRNAs that were strongly associated with GBM patients’ OS. We created a five-mRNA signature using multivariate Cox, LASSO estimation, and risk scoring techniques that were able to categorize GBM patients into a low-risk and high-risk group with significantly different OS. Since there is still the possibility of false positives from the development of the five-mRNA signature, we verified its predictive value using independent sets of different sample sizes (testing set, REMBRANDT study, CGGA datasets, and GSE7696). The results with the independent sets demonstrated that the five-mRNA signature has good reproducibility and robustness in predicting prognosis for GBM patients. Further analysis showed that the five-mRNA signature is independent of conventional clinical factors (age, gender, and KPS) and molecular features (IDH1 mutation and MGMT promoter methylation status). When we conducted a subgroup stratified analysis to test the signature’s independence, we discovered that the five-mRNA signature could clearly distinguish patients at low risk from those at high risk based on age, IDH1 wild-type, and methylated MGMT. Subsequently, we assessed the expression patterns of the mRNAs in the signature in specific subtypes of GBM (classical, mesenchymal, neural, and proneural) and discovered that there were significantly different expression patterns for all five prognostic mRNAs across the four GBM subtypes. These results indicated that the five-mRNA signature might help clinicians identify and select patients at high risk from those with identical clinical or molecular characteristics in order to rationalize treatment decisions.

Previous research has found that these five mRNAs are closely connected to the incidence and progression of tumors. Protein tyrosine phosphatase receptor type N (PTPRN), also recognized as IA-2, is a part of the protein tyrosine phosphatase (PTP) family, which includes signaling molecules that regulate a number of cellular processes such as cell growth, differentiation, the mitotic cycle, and oncogenic transformation (Alonso et al., 2016). Many members of this family have been reported to be closely related to tumors (Duś-Szachniewicz et al., 2015; Zhang et al., 2018; Bloch et al., 2019). PTPRN has been identified as an autoantigen that reacts with insulin-dependent diabetes mellitus (IDDM) patient sera (Lan et al., 1994; Acevedo-Calado et al., 2019). Only a few studies have reported a relationship with tumors, such as human midgut carcinoids and small cell lung cancer (Cunningham et al., 2000; Xu et al., 2016). Neuromedin B (NMB) is a member of the bombesin-like family of neuropeptides (Jensen et al., 2008). NMB functions by attaching to its high-affinity cell surface receptor, therefore activating multiple intracellular signaling pathways associated with cell proliferation, several anti-apoptotic genes, long-term memory, and learning. Park et al. (2016) reported that NMB receptor antagonism could inhibit the migration, invasion, and epithelial-mesenchymal transition of breast cancer cells. NMB also functions as an autocrine growth factor in lung cancer cells. The capacity of NMB to promote transactivation of the epidermal growth factor (EGF) receptor in lung cancer cells was observed by Moody et al. (2010). RALY RNA-binding protein-like (RALYL) is a protein-coding gene that may be involved in pre-mRNA splicing and embryonic development. Cui et al. (2012) discovered that low RALYL expression is linked to a poor prognosis in clear cell renal cell carcinoma. ATP-binding cassette subfamily C member 3 (ABCC3) is a member of the superfamily of ATP-binding cassette (ABC) transporters, which is implicated in multidrug resistance. ABCC3 knockdown may improve the retention of chemotherapeutic agents in breast cancer cells, making them more chemosensitive (Balaji et al., 2016). According to Liu et al. (2016), overexpression of ABCC3 enhances cell proliferation, drug resistance, and aerobic glycolysis, and is linked with a poor prognosis in patients with urinary bladder cancer. Midkine (MDK), also termed neurite growth-promoting factor 2, is a heparin-binding growth factor that is highly activated during oncogenesis, inflammation, and tissue repair. Recent studies indicate that serum MDK is a biomarker for malignancy, prognosis, and chemosensitivity in head and neck squamous cell carcinoma (Yamashita et al., 2016). Luo et al. (2015) showed that the transcriptional factor specificity protein 1 (SP1) enhances glioma cell proliferation by upregulating MDK. However, only a few reports have examined the correlation between GBM and the expression of the aforementioned mRNAs. Thus, we further investigated the potential functions of these five mRNAs in GBM using GSEA. The results showed that these five mRNAs may serve as oncogenes in GBM by regulating the cell cycle and cell apoptosis, brain development, immune response, MAPK signaling pathway, and ERBB signaling pathway. Moreover, we conducted GO and KEGG enrichment analyses of the encoded proteins that were co-expressed with these five mRNAs to investigate the roles of the five mRNAs. These five mRNAs’ potential protein targets were shown to play roles in neuron differentiation, brain development, the apoptosis pathway, the MAPK signaling pathway, the Ras signaling pathway, and the ERBB signaling pathway, according to GO and KEGG analyses. Based on the aforementioned bioinformatics analysis, we will pay more attention to the effects of immune microenvironment regulation, ERBB signaling pathway, and MAPK signaling pathway on the occurrence and development of GBM.

However, this study has a few limitations. Although we performed Cox proportional hazards regression analysis to explore the effect of age on the prognosis of GBM patients, we have not considered the influence of the age span. MGMT promote methylation and IDH-1 mutation were critical role in the prognosis of GMB patients (Zhou et al., 2019). However, there was still a lack of those research in the current studies. In order to further verify our bioinformatics predictions, there is a need for in-depth research on the five-mRNA signature and molecular mechanisms.

## Conclusion

In summary, we discovered a five-mRNA signature (PTPRN, NMB, RALYL, ABCC3, and MDK) among hundreds of potential mRNAs in large-scale GBM samples that can be employed as an independent prognostic marker in stratifying risk subgroups for GBM survival. This signature might also assist the researcher in better understanding the molecular mechanisms that contribute to the development of GBM. We will conduct further clinical trials to evaluate the signature’s predictive efficacy, and experimental research to examine the roles of the prognostic mRNAs.

## Data Availability

The original contributions presented in the study are included in the article/Supplementary Material; further inquiries can be directed to the corresponding author.

## References

[B1] Acevedo-Calado M. J. Pietropaolo S. L. Morran M. P. Schnell S. Vonberg A. D. Verge C. F. (2019). Autoantibodies Directed toward a Novel IA-2 Variant Protein Enhance Prediction of Type 1 Diabetes. Diabetes 68 (9), 1819–1829. 10.2337/db18-1351 31167877 PMC6702638

[B2] Alifieris C. Trafalis D. T. (2015). Glioblastoma Multiforme: Pathogenesis and Treatment. Pharmacol. Ther. 152, 63–82. 10.1016/j.pharmthera.2015.05.005 25944528

[B3] Alonso A. Nunes-Xavier C. E. Bayón Y. Pulido R. (2016). The Extended Family of Protein Tyrosine Phosphatases. Methods Mol. Biol. 1447, 1–23. 10.1007/978-1-4939-3746-2_1 27514797

[B4] Anjum K. Shagufta B. I. Abbas S. Q. Patel S. Khan I. Shah S. A. A. (2017). Current Status and Future Therapeutic Perspectives of Glioblastoma Multiforme (GBM) Therapy: A Review. Biomed. Pharmacother. 92, 681–689. 10.1016/j.biopha.2017.05.125 28582760

[B5] Balaji S. A. Udupa N. Chamallamudi M. R. Gupta V. Rangarajan A. (2016). Role of the Drug Transporter ABCC3 in Breast Cancer Chemoresistance. PLoS One 11 (5), e0155013. 10.1371/journal.pone.0155013 27171227 PMC4865144

[B6] Batash R. Asna N. Schaffer P. Francis N. Schaffer M. (2017). Glioblastoma Multiforme, Diagnosis and Treatment; Recent Literature Review. Curr. Med. Chem. 24 (27), 3002–3009. 10.2174/0929867324666170516123206 28521700

[B7] Bloch E. Sikorski E. L. Pontoriero D. Day E. K. Berger B. W. Lazzara M. J. (2019). Disrupting the Transmembrane Domain-Mediated Oligomerization of Protein Tyrosine Phosphatase Receptor J Inhibits EGFR-Driven Cancer Cell Phenotypes. J. Biol. Chem. 294 (49), 18796–18806. 10.1074/jbc.ra119.010229 31676686 PMC6901304

[B8] Bolstad B. M. Irizarry R. A. Astrand M. Speed T. P. (2003). A Comparison of Normalization Methods for High Density Oligonucleotide Array Data Based on Variance and Bias. Bioinformatics 19 (2), 185–193. 10.1093/bioinformatics/19.2.185 12538238

[B9] Cui Z.-W. Xia Y. Ye Y.-W. Jiang Z.-M. Wang Y.-D. Wu J.-T. (2012). RALY RNA Binding Protein-like Reduced Expression Is Associated with Poor Prognosis in Clear Cell Renal Cell Carcinoma. Asian Pac. J. Cancer Prev. 13 (7), 3403–3408. 10.7314/apjcp.2012.13.7.3403 22994768

[B10] Cunningham J. Lopez-Egido J. R. Janson E. T. Eriksson B. Oberg K. Gobl A. E. (2000). Transmembrane Protein Tyrosine Phosphatase IA-2 (ICA512) Is Expressed in Human Midgut Carcinoids but Is Not Detectable in Normal Enterochromaffin Cells. J. Endocrinol. 164 (3), 315–322. 10.1677/joe.0.1640315 10694371

[B11] Duś-Szachniewicz K. Woźniak M. Nelke K. Gamian E. Gerber H. Ziółkowski P. (2015). Protein Tyrosine Phosphatase Receptor R and Z1 Expression as Independent Prognostic Indicators in Oral Squamous Cell Carcinoma. Head. Neck 37 (12), 1816–1822. 10.1002/hed.23835 25043478

[B12] Fatai A. A. Gamieldien J. (2018). A 35-gene Signature Discriminates between Rapidly- and Slowly-Progressing Glioblastoma Multiforme and Predicts Survival in Known Subtypes of the Cancer. BMC Cancer 18 (1), 377. 10.1186/s12885-018-4103-5 29614978 PMC5883543

[B13] Han J. Puri R. K. (2018). Analysis of the Cancer Genome Atlas (TCGA) Database Identifies an Inverse Relationship between Interleukin-13 Receptor α1 and α2 Gene Expression and Poor Prognosis and Drug Resistance in Subjects with Glioblastoma Multiforme. J. Neurooncol. 136 (3), 463–474. 10.1007/s11060-017-2680-9 29168083 PMC5805806

[B14] Jensen R. T. Battey J. F. Spindel E. R. Benya R. V. (2008). International Union of Pharmacology. LXVIII. Mammalian Bombesin Receptors: Nomenclature, Distribution, Pharmacology, Signaling, and Functions in Normal and Disease States. Pharmacol. Rev. 60 (1), 1–42. 10.1124/pr.107.07108 18055507 PMC2517428

[B15] Lan M. S. Lu J. Goto Y. Notkins A. L. (1994). Molecular Cloning and Identification of a Receptor-type Protein Tyrosine Phosphatase, IA-2, from Human Insulinoma. DNA Cell Biol. 13 (5), 505–514. 10.1089/dna.1994.13.505 8024693

[B16] Leek J. T. Johnson W. E. Parker H. S. Jaffe A. E. Storey J. D. (2012). The Sva Package for Removing Batch Effects and Other Unwanted Variation in High-Throughput Experiments. Bioinformatics 28 (6), 882–883. 10.1093/bioinformatics/bts034 22257669 PMC3307112

[B17] Li X. Jin F. Li Y. (2021). A Novel Autophagy-Related lncRNA Prognostic Risk Model for Breast Cancer. J. Cell Mol. Med. 25 (1), 4–14. 10.1111/jcmm.15980 33216456 PMC7810925

[B18] Liu X. Yao D. Liu C. Cao Y. Yang Q. Sun Z. (2016). Overexpression of ABCC3 Promotes Cell Proliferation, Drug Resistance, and Aerobic Glycolysis and Is Associated with Poor Prognosis in Urinary Bladder Cancer Patients. Tumor Biol. 37 (6), 8367–8374. 10.1007/s13277-015-4703-5 26733163

[B19] Luo J. Wang X. Xia Z. Yang L. Ding Z. Chen S. (2015). Transcriptional Factor Specificity Protein 1 (SP1) Promotes the Proliferation of Glioma Cells by Up-Regulating Midkine (MDK). Mol. Biol. Cell 26 (3), 430–439. 10.1091/mbc.e14-10-1443 25428991 PMC4310735

[B20] Moody T. W. Berna M. J. Mantey S. Sancho V. Ridnour L. Wink D. A. (2010). Neuromedin B Receptors Regulate EGF Receptor Tyrosine Phosphorylation in Lung Cancer Cells. Eur. J. Pharmacol. 637 (1-3), 38–45. 10.1016/j.ejphar.2010.03.057 20388507 PMC3921891

[B21] Ostrom Q. T. Cioffi G. Gittleman H. Patil N. Waite K. Kruchko C. (2019). CBTRUS Statistical Report: Primary Brain and Other Central Nervous System Tumors Diagnosed in the United States in 2012-2016. Neuro Oncol. 21 (Suppl. 5), v1–v100. 10.1093/neuonc/noz150 31675094 PMC6823730

[B22] Park H.-J. Kim M.-K. Choi K.-S. Jeong J.-W. Bae S.-K. Kim H. J. (2016). Neuromedin B Receptor Antagonism Inhibits Migration, Invasion, and Epithelial-Mesenchymal Transition of Breast Cancer Cells. Int. J. Oncol. 49 (3), 934–942. 10.3892/ijo.2016.3590 27571778

[B23] Polivka J. Jr. Polivka J. Holubec L. Kubikova T. Priban V. Hes O. (2017). Advances in Experimental Targeted Therapy and Immunotherapy for Patients with Glioblastoma Multiforme. Anticancer Res. 37 (1), 21–33. 10.21873/anticanres.11285 28011470

[B24] Qian Z. Li Y. Ma J. Xue Y. Xi Y. Hong L. (2018). Prognostic Value of NUSAP1 in Progression and Expansion of Glioblastoma Multiforme. J. Neurooncol. 140 (2), 199–208. 10.1007/s11060-018-2942-1 29995176

[B25] Sasmita A. O. Wong Y. P. Ling A. P. K. (2018). Biomarkers and Therapeutic Advances in Glioblastoma Multiforme. Asia-Pac J. Clin. Oncol. 14 (1), 40–51. 10.1111/ajco.12756 28840962

[B26] Stein C. K. Qu P. Epstein J. Buros A. Rosenthal A. Crowley J. (2015). Removing Batch Effects from Purified Plasma Cell Gene Expression Microarrays with Modified ComBat. BMC Bioinforma. 16, 63. 10.1186/s12859-015-0478-3 PMC4355992 25887219

[B27] Verhaak R. G. W. Hoadley K. A. Purdom E. Wang V. Qi Y. Wilkerson M. D. (2010). Integrated Genomic Analysis Identifies Clinically Relevant Subtypes of Glioblastoma Characterized by Abnormalities in PDGFRA, IDH1, EGFR, and NF1. Cancer Cell 17 (1), 98–110. 10.1016/j.ccr.2009.12.020 20129251 PMC2818769

[B28] Xu H. Cai T. Carmona G. N. Abuhatzira L. Notkins A. L. (2016). Small Cell Lung Cancer Growth Is Inhibited by miR-342 through its Effect of the Target Gene IA-2. J. Transl. Med. 14 (1), 278. 10.1186/s12967-016-1036-0 27670444 PMC5037891

[B29] Yamashita T. Shimada H. Tanaka S. Araki K. Tomifuji M. Mizokami D. (2016). Serum Midkine as a Biomarker for Malignancy, Prognosis, and Chemosensitivity in Head and Neck Squamous Cell Carcinoma. Cancer Med. 5 (3), 415–425. 10.1002/cam4.600 26798989 PMC4799940

[B30] Yi H. Raman A. T. Zhang H. Allen G. I. Liu Z. (2018). Detecting Hidden Batch Factors through Data-Adaptive Adjustment for Biological Effects. Bioinformatics 34 (7), 1141–1147. 10.1093/bioinformatics/btx635 29617963 PMC6454417

[B31] Zhang G. Liu X. Wu J. Zhu Q. Zeng H. Wang T. (2018). Expression and Clinical Relations of Protein Tyrosine Phosphatase Receptor Type S in Esophageal Squamous Cell Carcinoma. Histol. Histopathol. 33 (11), 1181–1188. 10.14670/HH-18-002 29745967

[B32] Zhou M. Niu C. Jia L. He H. (2019). The Value of MGMT Promote Methylation and IDH-1 Mutation on Diagnosis of Pseudoprogression in Patients with High-Grade Glioma: A Meta-Analysis. Med. Baltim. 98 (50), e18194. 10.1097/MD.0000000000018194 PMC6922590 31852075

