# Supplementary material for: Identification of a Five-mRNA Signature as a Novel Potential Prognostic Biomarker for Glioblastoma by Integrative Analysis
Source: Front Genet. 2022 Jul 8;13:931938. doi: 10.3389/fgene.2022.931938 (PMC9305328; doi:10.3389/fgene.2022.931938)
Supplement: Supplementary file 1 [file DataSheet1.DOCX]

**Supplementary material**

**
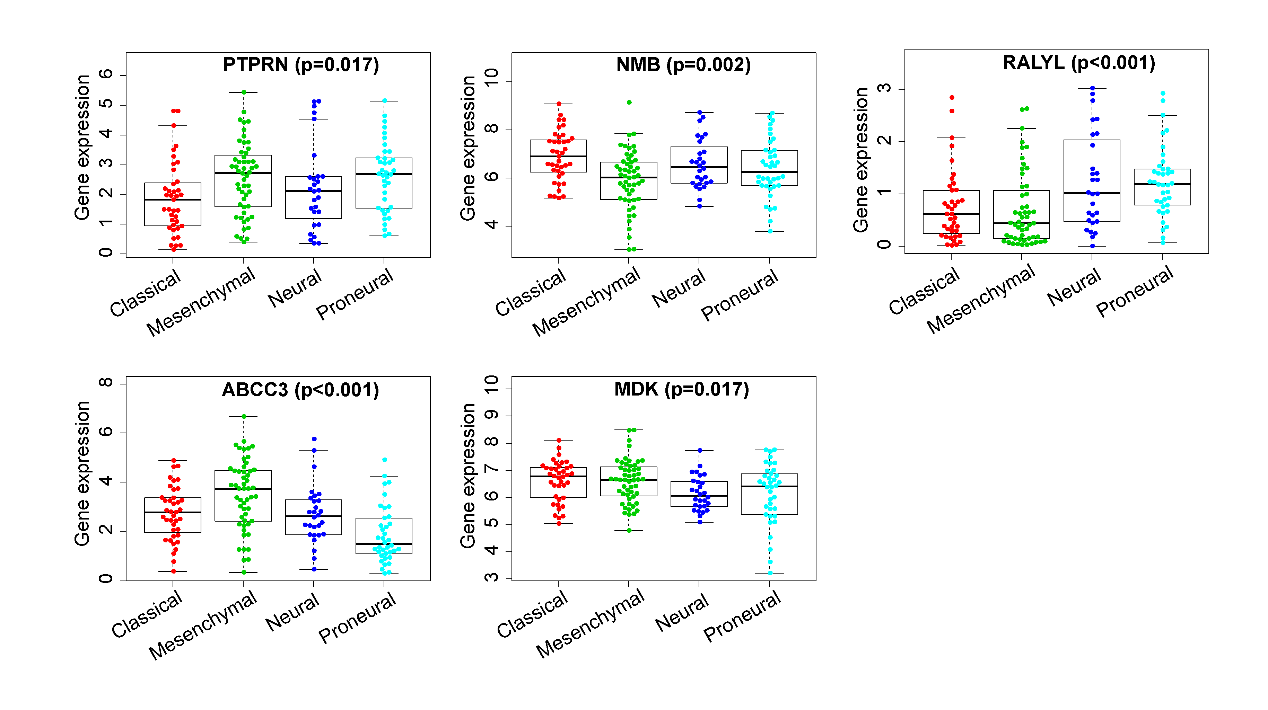
**

**Figure S1.** The expression levels of the five-mRNA signature in the four subtypes of GBM.

**Table S1**. The sequences of the specific primers

| **Gene** | **Primers** | |
| --- | --- | --- |
| PTPRN | Forward | 5’-CATACCATCGCAGACTTCT-3’ |
|  | Reverse | 5’-GCACTTGTTCACCTTCCT-3 |
| NMB | Forward | 5’-AACAGCGTGGCTTAGATT-3’ |
|  | Reverse | 5’-CACAGTAATGGAGTAACAGAG-3’ |
| RALYL | Forward | 5’-CTCCTCCTCTTCGCAATG-3’ |
|  | Reverse | 5’-CGCTCACTCATGTACTGTA-3’ |
| ABCC3 | Forward | 5’-GCAGACAGTAGACAGAACA-3’ |
|  | Reverse | 5’-AACCTCATCAACGACATAGA-3’ |
| MDK | Forward | 5’-GCAACTGGAAGAAGGAGT-3’ |
|  | Reverse | 5’-TGGCACTGAGCATTGTAG-3’ |
| GAPDH | Forward | 5’-TGAAGGTCGGAGTCAACGG-3’ |
|  | Reverse | 5’-CCTGGAAGATGGTGATGGG-3’ |
